# Melatonin Promotes *in vitro* Development of Vitrified-Warmed Mouse GV Oocytes, Potentially by Modulating Phosphorylation of Drp1

**DOI:** 10.3389/fvets.2021.752001

**Published:** 2021-09-24

**Authors:** Jianpeng Qin, Shichao Guo, Jinyu Yang, Izhar Hyder Qazi, Bo Pan, Tianyi Lv, Shengqin Zang, Yi Fang, Guangbin Zhou

**Affiliations:** ^1^Department of Animal Science, Farm Animal Genetic Resources Exploration and Innovation Key Laboratory of Sichuan Province, College of Animal Science and Technology, Sichuan Agricultural University, Chengdu, China; ^2^Department of Veterinary Anatomy and Histology, Shaheed Benazir Bhutto University of Veterinary and Animal Sciences, Sakrand, Pakistan; ^3^Department of Grassland Resources and Animal Husbandry, Jilin Provincial Key Laboratory of Grassland Farming, Northeast Institute of Geography and Agoecology, Chinese Academy of Sciences, Changchun, China

**Keywords:** melatonin, oocyte cryopreservation, mitochondrial, drp1 phosphorylation, apoptosis

## Abstract

Previous studies have shown that melatonin can mitigate cryopreservation-induced mitochondrial dysfunction in oocytes; however, the underlying molecular mechanism remains unclear. The objective of the present study was to investigate whether melatonin can improve the mitochondrial function during *in vitro* maturation of vitrified-warmed mouse germinal vesicle (GV) oocytes by modulating phosphorylation of dynamin related protein 1 (Drp1). Vitrification/warming procedures resulted in the following: (1) After cryopreservation of mouse GV oocytes, the phosphorylation level of Drp1 at Ser616 (p-Drp1 Ser616) in metaphase II (MII) oocytes was increased (*P* < *0.05*). Furthermore, the rates of *in vitro* maturation, cleavage and blastocyst formation after parthenogenetic activation were decreased (*P* < *0.05*). (2) In MII oocytes, the expression levels of translocase of the mitochondrial outer membrane 20 (TOMM20), mitochondrial membrane potential (MMP), adenosine triphosphate (ATP) content, and mRNA levels of mitochondrial biogenesis-related genes (*Sirt1, Pgc-1*α*, Tfam*) were all decreased (*P* < *0.05*), and (3) Reactive oxygen species (ROS) level, early apoptosis level, Cytochrome C release and mRNA levels of pro-apoptotic related genes (*Bax, Caspase9, Caspase3*) in MII oocytes were all increased (*P* < *0.05*). The results of this study further revealed that negative impacts of GV oocyte cryopreservation were mitigated by supplementation of warming and *in vitro* maturation media with 10^−7^mol /L melatonin or 2 x 10^−5^mol/L Mdivi-1 (Drp1 inhibitor). Therefore, we concluded that 10^−7^mol/L melatonin improved mitochondrial function, reduced oxidative stress and inhibited apoptosis by regulating phosphorylation of Drp1, thereby enhancing *in vitro* development of vitrified-warmed mouse GV oocytes.

## Introduction

In recent years, cryopreservation of oocytes has been widely applied in preservation of animal germplasm, developmental biology research, and assisted reproduction among others (1–3). In animal production, this approach could be used to establish oocyte banks, facilitating animal production and breeding programs (4), and also preserving diversity of livestock genetic resources (5–8). In human reproductive medicine, this can facilitate treatment of female infertility resulting from cancer therapy and premature ovarian failure (9–11), and would avoid many ethical, legal, moral and religious issues of embryo freezing (12, 13). However, oocyte cryopreservation can cause mitochondrial function damage and produce excessive ROS, with deleterious effects on oocyte development (14–16). Therefore, reducing mitochondrial damage is one approach to improve the developmental potential of vitrified-warmed oocytes.

Under normal physiological conditions, mitochondrial homeostasis is precisely regulated by mitochondrial fission and fusion to meet physiological needs (17, 18). Among them, mitochondrial fission is mainly regulated by the dynamin related protein 1 (Drp1) (19). The phosphorylation of Drp1 at serine 616 increases its activity (20). Activated Drp1 is recruited to the mitochondrial outer membrane by the protein receptor on the mitochondrial outer membrane (21), and Drp1 comprises the helical oligomer around the mitochondrial outer membrane to drive the fission process (22). Abnormal expression and activity of Drp1 could cause an imbalance of mitochondrial fission/fusion, potentially affecting mitochondrial function and ultimately leading to apoptosis (23, 24). When p-Drp1 Ser616 increases in SD rat cardiac myocytes, excessive fission of mitochondria occurred, leading to cardiac dysfunction (23). Inhibition of Drp1 activity affects mitochondrial function and increases oxidative stress and apoptosis during maturation of porcine oocytes (25). In addition, maintenance of mitochondrial function by p-Drp1 Ser616 was observed in mouse oocytes (26). However, it is still unknown whether oocyte cryopreservation alters Drp1 expression and p-Drp1 Ser616 during *in vitro* maturation of mouse GV oocytes.

Mitochondrial biogenesis contributes to maintenance of mitochondrial homeostasis in cells (27), which is regulated by many factors (28). Among them, sirtuin 1 (Sirt1) is a member of the deacetylase family that is involved in mitochondrial biogenesis and energy metabolism (29). Peroxisome proliferator activated receptor gamma coactivator alpha (Pgc-1α) and mitochondrial transcription factor A (Tfam), key factors in mitochondrial biogenesis, could regulate transcription and replication of the mitochondrial genome (30). Cryopreservation of porcine GV oocytes and 2-cell mouse embryos can cause expression disorder of *Sirt1, Pgc-1*α and *Tfam*, resulting in mitochondrial dysfunction and disrupting cell development (15, 16). TOMM20 is an important indicator of mitochondrial mass and metabolic activity (31). Cryopreservation of porcine GV oocytes decreased expression of TOMM20 and caused mitochondrial damage (16). To sum up, abnormal expression of mitochondrial biogenesis related genes (*Sirt1, Pgc-1*α and *Tfam*) and TOMM20 could affect mitochondrial function and block cell development. However, it is yet to be determined whether cryopreservation could alter mitochondrial biogenesis mentioned above during *in vitro* maturation of vitrified-warmed GV mouse oocytes.

Melatonin is secreted predominantly in the pineal gland and has many important biological functions (32). Melatonin can inhibit excessive fission of mitochondria by down-regulating expression of Drp1 to reduce the damage caused by calcification of rat vascular smooth muscle cells (24). It can also inhibit rotenone-induced SH-SY5Y cell death (33), maintain cardiac function in rats with mechanical trauma (23), protect cardiac microvasculature from ischemia/reperfusion injury (34) and treat prion disease (35) by regulating Drp1 activity. Furthermore, melatonin can reduce cryopreservation-induced mitochondrial damage, oxidative stress, and apoptosis in mouse (2, 36–38), horse (7), cattle (14) and human (39) oocytes. However, it remains to be explored whether melatonin involvement in the above-mentioned functions during *in vitro* development of vitrified-warmed mouse GV oocytes occurs via regulation of Drp1 or p-Drp1 Ser616.

To elucidate potential mechanisms of melatonin in mitochondrial function, we conducted the following experiments. (1) Effects of melatonin on both Drp1 and p-Drp1 Ser616 during *in vitro* maturation of vitrified-warmed mouse GV oocytes and their developmental potential (e.g., rates of maturation, cleavage and blastocyst formation). (2) Effects of melatonin on mitochondrial mass (TOMM20) and mitochondrial function (MMP, ATP, mitochondrial biogenesis related genes *Sirt1, Pgc-1*α, *Tfam*) through regulation of p-Drp1 Ser616 during *in vitro* maturation of vitrified-warmed mouse GV oocytes. and 3) Effects of melatonin on oxidative stress (ROS and Cytochrome C) and apoptosis (level of early apoptosis, pro-apoptotic related genes *Bax, Caspase9, Caspase3*) through regulation p-Drp1 Ser616 during *in vitro* maturation of vitrified-warmed mouse GV oocytes.

## Materials and Methods

Unless otherwise stated, all chemicals were purchased from Sigma-Aldrich (St. Louis, MO, USA). All experimental procedures were conducted in strict accordance with regulations of the animal ethical and welfare committee (AEWC) of Sichuan Agricultural University China (approval code: AEWC2016, 6 January 2016).

### Oocyte Collection

Female ICR mice, 8–10 wk old, were purchased from Chengdu Dashuo Experimental Animal Co., Ltd, and maintained at 18–25°C and 50–70% humidity, with 14 h of light and 10 h of darkness. After a 2-wk adaptation period, each mouse was given 10 IU PMSG (PMSG, Ningbo Shusheng Veterinary Drug Co., Ltd, Ningbo, China) ip. After 44–48 h, the mice were euthanized by cervical dislocation. Ovaries were removed, placed in a 37°C M2 solution, sliced under the stereomicroscope with syringe needles, and GV oocytes with obvious germinal vesicles were selected for experiments.

### Oocyte Vitrification and Warming

Oocytes were vitrified using an open-pulled straws (OPS) method, as described in our previous study (40). Briefly, the straws (250 μL, IMV, France) were heat-softened and pulled manually, to obtain straws ~ 3 cm long, 0.10 mm inner diameter, and 0.15 mm outer diameter.

Vitrification-warming procedures were conducted in accordance with our standard laboratory practice (2). Briefly, oocytes were first equilibrated in 10% ethylene glycol (EG) +10% dimethyl sulfoxide (DMSO) for 30 s, then loaded into the narrow end of OPS with EDFS30 solution, comprised of DPBS medium containing 300 g/L Ficoll, 0.5 mol/L sucrose, and 3 g/L bovine serum albumin (BSA), 15% EG and 15% DMSO, for 25 s. Finally, straws containing oocytes were plunged into liquid nitrogen. During warming, oocytes were rinsed in 0.5 mol/L sucrose for 5 min, and then washed three times in M2 medium. All manipulations were performed at 37°C on a warming stage attached to a stereomicroscope (SMZ1500, Nikon, Tokyo, Japan).

### Oocyte Culture and *in vitro* Maturation

The GV oocytes were randomly divided into four groups. Fresh group (F): Fresh oocytes matured directly *in vitro*. Vitrification group (V): Oocytes were vitrified and then matured *in vitro*. Vitrification + Melatonin group (V+M): On the basis of vitrification group, melatonin [10^−7^mol /L, concentration based on our previous study (2)] was added to the warming and maturation solutions (M16); Vitrification + Mdivi-1 group (V+MD, Mdivi-1: 14102, HY15886, Med Chem Express, Shanghai, China): On the basis of vitrification group, Mdivi-1 [2x10^−5^mol/L, concentration based on a previous study (41)] was added to the warming and maturation solutions. Then, fresh and vitrified oocytes were rinsed and placed in M16 medium. Metaphase I (MI) and metaphase II (MII) oocytes were collected after 8 and 12 h of *in vitro* culture of GV oocytes, respectively. Maturation rates in each group were calculated.

### Parthenogenetic Activation and Embryo Culture

Methods for parthenogenetic activation of oocytes and *in vitro* culture of resultant embryos were adopted as described in a previous study (36). Briefly, all *in vitro* matured oocytes were incubated first in activation solution A (Ca^2+^-free human tubal fluid (HTF) supplemented with 10 mmol/L SrCl_2_ and 2 mg/mL cytochalasin D) for 2.5 h and then in activation solution B (HTF supplemented with 2 mg/mL cytochalasin D) for 3.5 h at 37.5°C in a humidified atmosphere with 5% CO_2_ in air. Finally, oocytes were removed from above media and cultured in KSOM-AA medium. Cleavage and blastocyst rates were calculated at 24 and 96 h post activation.

### Detection of MMP and ATP Level

MMP was detected by JC-1 staining according to manufacturer's guidelines (Beijing Solarbio Science & Technology Co., Ltd., Beijing, China). Firstly, JC-1 probe was diluted to a final concentration of 10 μg/mL in M2 solution and then equilibrated in a humidified incubator containing 5% CO_2_ at 37°C for 20 min. Secondly, all oocytes were stained in a humidified incubator containing 5% CO_2_ at 37°C for 15 min. The JC-1 reaction was conducted in darkness. Then, oocytes were washed three times for 5 min each in M2 solution without JC-1 probe. Finally, they were transferred to a slide containing VECTASHIELD mounting medium with DAPI and sealed with a glass cover and photographed under fluorescence microscopy (BX53, Olympus, Tokyo, Japan). The ratio of red fluorescence to green fluorescence was recorded as MMP (Δψm) of oocytes. The intensity of red and green fluorescence in each oocyte was measured using Image J software (Version 1.48; National Institutes of Health, Bethesda, MD, USA), calculated the fluorescence intensity was based on previous study (2, 38, 42).

ATP levels were determined according to the manufacturer's instructions (A095-2, Nanjing Jiancheng Bioengineering Institute, Nanjing, China). Firstly, oocytes were initially washed three times with M2 and added to an Eppendorf tube containing 20 μL of ATP lysate for ATP detection (groups of 10 oocytes each). Secondly, the enzyme working solution was configured and samples were treated according to the instructions. ATP levels were measured using a multi-plate reader containing chemiluminescence (Varioskan LUX, Thermo, USA). Finally, sample ATP concentration was calculated using a standard curve generated from nine ATP gradient concentrations ranging from 0 to 2 μmol/L.

### Detection of ROS Level and Early Apoptosis

To measure intracellular ROS level, oocytes were incubated in M2 solution containing 20 μM 2, 7-dichlorodihydrofluorescein diacetate (H2DCFDA, C2938, Invitrogen, Carlsbad, CA, USA) for 30 min (37°C, 100% humidity, and 5% CO_2_), and then washed three times in M2 solution for 5 min each. Finally, oocytes were placed on a clean glass slide and photographed under fluorescence microscopy and fluorescence measured, as described above.

Similarly, according to the manufacturer's guidelines of Annexin-V kit (C1062L, Beyotime Biotechnology, Shanghai, China), oocytes were collected and transferred to a working solution of Annexin-V. After 30 min incubation at 37°C, oocytes were washed three times in M2 solution for 5 min each. At the end, oocytes were transferred to slide containing VECTASHIELD mounting medium with DAPI and sealed with glass cover and photographed under a fluorescence microscope and the intensity of fluorescence in each oocyte was measured as described above.

### Immunofluorescent Staining

Oocytes were fixed in 4% paraformaldehyde for 30 min, and then permeabilized in permeate (DPBS containing 1% Triton X-100) for 20 min at room temperature. After blocking oocytes with 1% BSA for 1 h at room temperature, they were exposed to primary antibody at 4°C overnight, and washed three times for 10 min each in wash buffer (DPBS containing 0.01% Triton X-100 and 0.1% Tween 20), then stained with fluorescently labeled secondary antibodies and incubated at 37°C for 1 h. After incubation, oocytes were washed three times in washing buffer for 10 min each. Finally, oocytes were transferred to slide containing VECTASHIELD mounting medium with DAPI and sealed with glass cover and photographed under fluorescence microscope and intensity measured as described above.

Antibodies and dilution ratios used in immunofluorescence were as follows: Drp1 antibody (Affinity, DF7037, 1:300); Drp1(Ser616) antibody (Affinity, AF8470, 1:300); TOMM20 antibody (Affinity, DF4179, 1:200); Cytochrome C antibody (Affinity, AF0146, 1:100); CoraLite488- conjugated Goat Anti-Rabbit IgG (Proteintech, SA00013-2, 1:300).

### Quantitative Polymerase Chain Reaction (Q-PCR)

The total cDNA was isolated from oocytes (≧20) using TransScript-Uni Cell to cDNA Synthesis SuperMix for Q-PCR kit (TransGen Biotech, Beijing, China). Then, cDNA was quantified by Q-PCR using a TransStart Tip Green qPCR SuperMix Kit (TransGen Biotech, Beijing, China) on a CFX Connect Real-Time Detection System (Bio-Rad, Hercules, CA, USA) under standard conditions. The cycle threshold (Ct) value used to calculate the relative expression was the average of three replicates and was normalized against that of the reference gene (*Gapdh*). In this experiment, mitochondrial biogenesis related genes (*Sirt1, Pgc-1*α, *Tfam*) and pro-apoptotic related genes (*Bax, Caspase9, Caspase3*) were detected in MII oocytes. Primer information is summarized in Table 1. The mRNA expression levels were calculated using the 2^−ΔΔCt^ method.

**Table 1:** The specific primers used for Q-PCR in this study.

**Genes**	**GenBank number**	**Primer sequences**	**Tm (^**°**^C)**
*Sirt1*	NM_019812.3	F: TCGTGGAGACATTTTTAATCAGGR: GCTTCATGATGGCAAGTGG	55
*Pgc-1α*	NM_008904.2	F: AGAACGTGACCTTATCACCCCR: GCACCTCAACCCGACTACTT	55
*Tfam*	NM_009360.4	F: GTGAGCAAGTATAAAGAGCAGCR: CTGAACGAGGTCTTTTTGGTTT	55
*Bax*	NM_007527.3	F: ATGCGTCCACCAAGAAGC	55
		R: CCAGTTGAAGTTGCCATCAG	
*Caspase9*	NM_015733.5	F: TGTGAATATCTTCAACGGGAGC	55
		R: GAGTAGGACACAAGGATGTCAC	
*Caspase3*	NM_009810.3	F: AAAGGCTGGAACCCTTGTTT	55
		R: GCACCTTGCCTTCAATGAGT	
*Gapdh*	NM_008084.3	F: CATGGCCTTCCGTGTTCCTAR: GCCTGCTTACCACCTTCTT	55

*Tm, Melting temperature; Sirt1, Sirtuin 1; Pgc-1α, Peroxisome proliferator activated receptor gamma coactivator alpha; Tfam, Mitochondrial transcription factor A; Bax, B-cell lymphoma-2 associated X; Caspase, Cysteinyl aspartate specific proteinase; Gapdh, Glyceraldehyde-3-phosphate dehydrogenase*.

### Experimental Design

**Figure 1:**
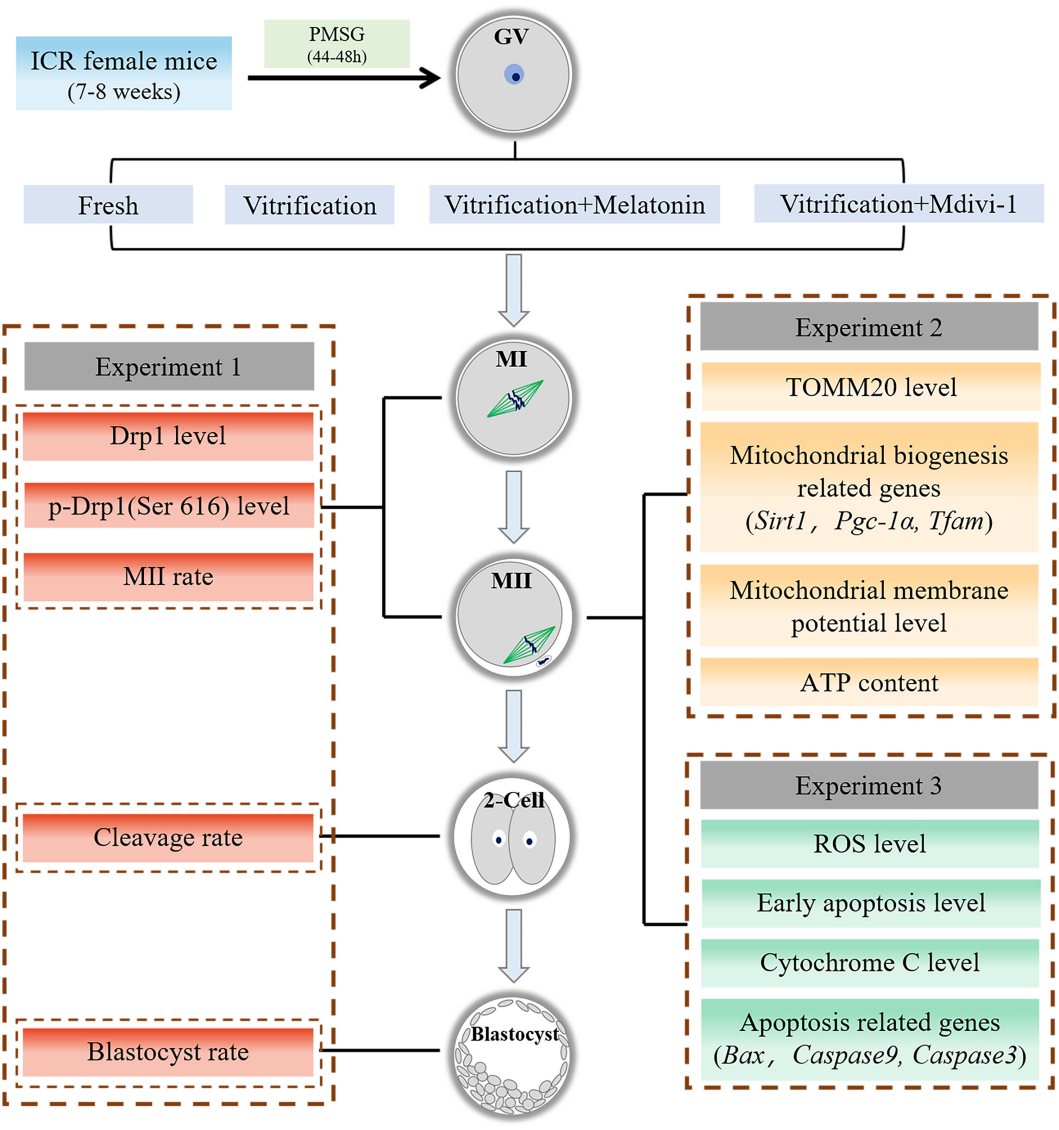
Experiment flow chart. GV, germinal vesicle; MI, metaphase I; MII, metaphase II. Melatonin and Mdivi-1 were added to warming and maturation solutions.

The outline for the design of the experiments is presented in Figure 1. This study consisted of Experiments 1, 2, and 3. In each experiment, all fresh GV oocytes were randomly divided into four groups: Fresh group, Vitrification group, Vitrification + Melatonin group and Vitrification + Mdivi-1 group.

Experiment 1: Effects of melatonin on Drp1 and developmental potential during *in vitro* maturation of vitrified-warmed mouse GV oocytes. (1) Expression level of Drp1 and level of p-Drp1 Ser616 were detected in MI and MII oocytes by immunofluorescence staining. (2) Maturation rate, cleavage rate at 24 h and blastocyst rate at 96 h after parthenogenetic activation were calculated in each group.

Experiment 2: Effects of melatonin on mitochondrial mass and mitochondrial function in vitrified-warmed mouse GV oocytes through regulation of p-Drp1 Ser616. (1) Expression level of TOMM20 and MMP were detected in MII oocytes by immunofluorescence staining, and ATP content was measured using a multi-plate reader containing chemiluminescence in MII oocytes. (2) Levels of mRNA of mitochondrial biogenesis related genes (*Sirt1, Pgc-1*α, *Tfam*) were detected in MII oocytes with Q-PCR.

Experiment 3: Effects of melatonin on oxidative stress and apoptosis in vitrified-warmed mouse GV oocytes through regulation of p-Drp1 Ser616. (1) The ROS level, early apoptosis and Cytochrome C content were detected in MII oocytes by immunofluorescence staining. (2) Levels of mRNA of pro-apoptotic related genes (*Bax, Caspase9, Caspase3*) were detected in MII oocytes with Q-PCR.

### Statistical Analyses

Statistical analyses were performed using one-way ANOVA, followed by a *post-hoc* Fisher's least significant difference (LSD) test, using SPSS statistical software (v. 22.0; IBM, Chicago, IL, USA). Normality test and variance homogeneity test were used before ANOVA. Prior to ANOVA, percentage data were arcsine transformed. Data were expressed as the mean ± standard error and all experiments were repeated at least three times. For all analyses, *P* < *0.05* was regarded as significant.

## Results

### Melatonin Regulated the LEVEL of p-Drp1 Ser616 During *in vitro* Maturation of Vitrified-Warmed Mouse GV Oocytes

**Figure 2:**
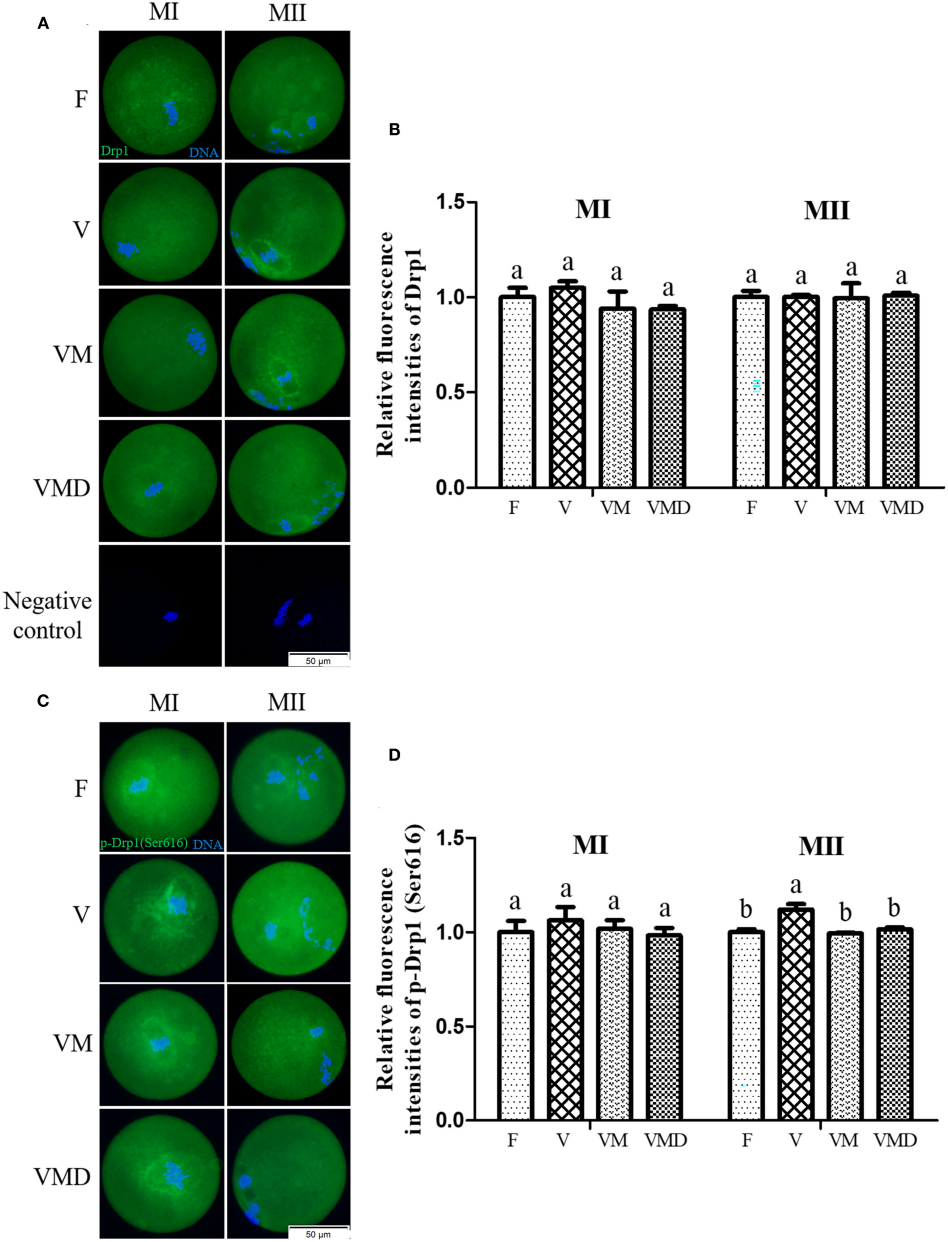
Effects of melatonin on Drp1 activity during *in vitro* culture of vitrified-warmed mouse GV oocytes. **(A)** Representative images of Drp1 in mouse MI and MII oocytes. Scale bar, 50 μm. **(B)** The fluorescence intensity of Drp1 was recorded using ImageJ software. In total, *n* = 174 MI oocytes (F: *n* = 45; V: *n* = 42; VM: *n* = 45; VMD: *n* = 42) and *n* = 147 MII oocytes (F: *n* = 39; V: *n* = 40; VM: *n* = 35; VMD: *n* = 33) were used in this assay. **(C)** Representative images of p-Drp1 (Ser616) in mouse MI and MII oocytes. Scale bar, 50 μm. **(D)** Fluorescence intensity of p-Drp1 (Ser616) was recorded using ImageJ software. In total, *n* = 154 MI oocytes (F: *n* = 36; V: *n* = 40; VM: *n* = 35; VMD: *n* = 43) and *n* = 150 MII oocytes (F: *n* = 40; V: *n* = 38; VM: *n* = 40; VMD: *n* = 32) were used in this assay. Data are presented as mean ± SEM of three independent experiments. Groups without a common superscript are significantly different (*P* < *0.05*). The four experimental groups: Fresh control (F), vitrification (V), vitrification + MT (VM), and vitrification + Mdivi-1 (VMD). MI, metaphase I; MII, metaphase II.

Expression level of Drp1, represented by relative fluorescence intensity, in both MI and MII oocytes derived from vitrified-warmed mouse GV oocytes were similar (*P* > *0.05*) to that of corresponding fresh group (Figures 2A,B). When melatonin or Mdivi-1 were added in the vitrification group, Drp1 expression in both MI and MII oocytes were not different (*P* > *0.05*) compared to that of corresponding vitrification and fresh groups, respectively. Furthermore, p-Drp1 Ser616 expression, represented by relative fluorescence intensity (Figures 2C,D), in MI oocytes, was not different (*P* > *0.05*) among F, V, VM, and VMD groups. However, p-Drp1 Ser616 expression in MII oocytes was higher (*P* < *0.05*) in the vitrification group than in the fresh group. When melatonin or Mdivi-1 were added to warming and maturation solutions, p-Drp1 Ser616 expression in MII oocytes was lower (*P* < *0.05*) than that of vitrification group, but similar (*P* > *0.05*) to the fresh group.

### Melatonin Promotes *in vitro* Development of Vitrified-Warmed Mouse GV Oocytes by Regulating p-Drp1 Ser616

**Figure 3:**
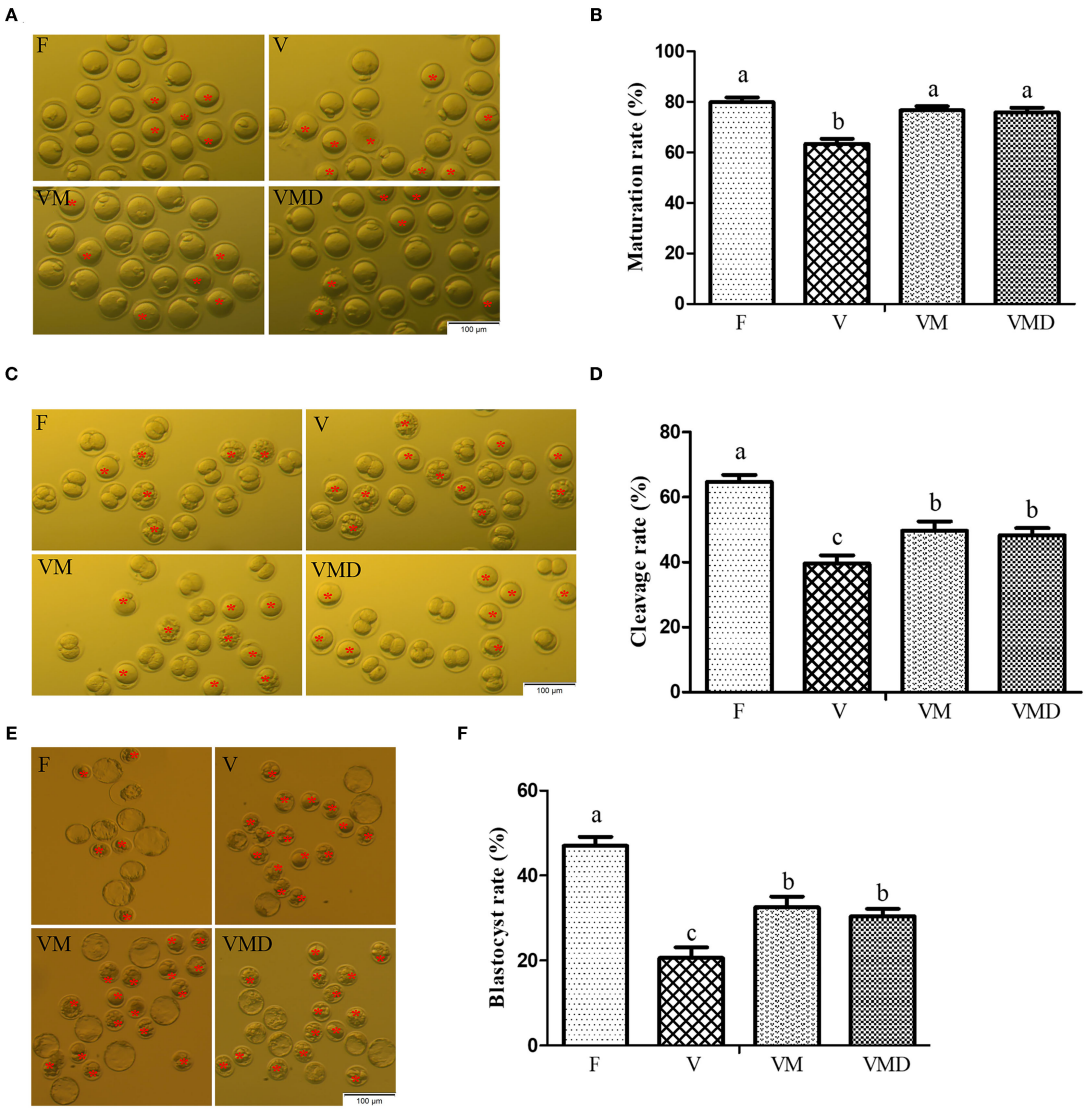
Effects of melatonin on *in vitro* developmental potential of vitrified-warmed mouse GV oocytes. **(A)** Representative images of matured oocytes cultured *in vitro*. Scale bar, 100 μm. **(B)** The maturation rate was recorded in F (*n* = 179), V (n = 184), VM (*n* = 179), and VMD (*n* = 194) oocytes after maturation for 12 h *in vitro*. Data are presented as mean ± SEM of seven independent experiments. **(C)** Representative images of 2-Cell from different groups. Scale bar, 100 μm. **(D)** The cleavage rate was recorded in F (*n* = 51), V (*n* = 43), VM (*n* = 54) and VMD (*n* = 53) 2-Cell after parthenogenetic activation for 24 h. Data are presented as mean ± SEM of five independent experiments. **(E)** Representative images of blastocysts. Scale bar, 100 μm. **(F)** The blastocyst rate was recorded in F (*n* = 37), V (*n* = 22), VM (*n* = 35) and VMD (*n* = 33) blastocysts after parthenogenetic activation for 96 h. Data are presented as mean ± SEM of five independent experiments. Groups without a common superscript are significantly different (*P* < *0.05*). The four experimental groups: Fresh control (F), vitrification (V), vitrification + MT (VM), and vitrification + Mdivi-1 (VMD). The cleavage rate and blastocyst rate were calculated on the number of mature oocytes. The red asterisk indicates cell development retardation.

At 12 h after *in vitro* culture of mouse GV oocytes, the maturation rate was lower (*P* < *0.05*) in the vitrification group (63.35 ± 2.03%) than in the fresh group (79.90 ± 1.90%) (Figures 3A,B). However, when melatonin or Mdivi-1 were added, maturation rates of vitrified-warmed oocytes were 76.59 ± 1.69 and 75.88 ± 1.84%, respectively, higher (*P* < *0.05*) than in the corresponding vitrification group, but not different (*P* > *0.05*) from the fresh group. The cleavage rate was lower (*P* < *0.05*) in the vitrification group (39.60 ± 2.53%) than in the fresh group (64.69 ± 2.21%) at 24 h after parthenogenetic activation (Figures 3C,D). When melatonin or Mdivi-1 was added, cleavage rates of vitrified-warmed oocytes were 49.72 ± 2.84 and 48.32 ± 2.20%, respectively, higher (*P* < *0.05*) than in the corresponding vitrification group, but lower (*P* < *0.05*) than in the corresponding fresh group. The blastocyst rate was lower (*P* < *0.05*) in the vitrification group (20.64 ± 2.50%) than in the fresh group (47.02±2.12%) at 96 h after parthenogenetic activation (Figures 3E,F). When melatonin or Mdivi-1 was added, cleavage rates of vitrified-warmed oocytes were 32.52 ± 2.54 and 30.43 ± 1.74%, respectively, higher (*P* < *0.05*) than in the corresponding vitrification group, and lower (*P* < *0.05*) than in the corresponding fresh group.

### Melatonin Alleviates Mitochondrial Dysfunction Caused by Cryopreservation of Mouse GV Oocytes via Regulating p-Drp1 Ser616

**Figure 4:**
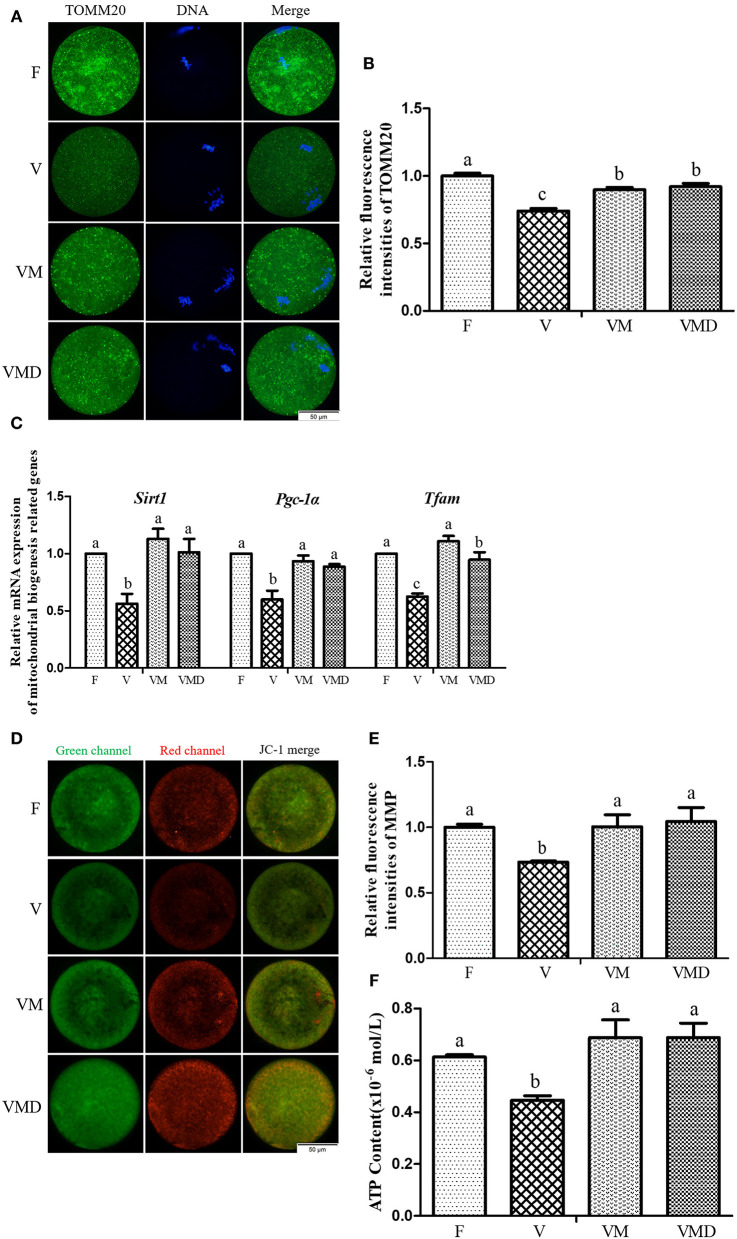
Effects of melatonin on mitochondrial mass and mitochondrial function in MII oocytes derived from vitrified-warmed mouse GV oocytes. **(A)** Representative images of TOMM20 in mouse MII oocytes. Scale bar, 50 μm. **(B)** The fluorescence intensity of TOMM20 was recorded using ImageJ software. In total, *n* = 197 MII oocytes were used in this assay (F: *n* = 53; V: *n* = 49; VM: *n* = 48; VMD: *n* = 47). **(C)** The expression levels of mitochondrial biogenesis related genes. **(D)** Representative images of mitochondrial membrane potential (MMP) in mouse MII oocytes. Scale bar, 50 μm. **(E)** The rate of mitochondrial membrane potential was recorded using ImageJ software. In total, *n* = 129 MII oocytes were used in this assay (F: *n* = 32; V: *n* = 27; VM: *n* = 36; VMD: *n* = 34). **(F)** The ATP content in different groups. In total, *n* = 120 MII oocytes were used in this assay (F: *n* = 30; V: *n* = 30; VM: *n* = 30; VMD: *n* = 30). Data are presented as mean ± SEM of three independent experiments. Groups without a common superscript are significantly different (*P* < *0.05*). The four experimental groups: Fresh control (F), vitrification (V), vitrification + MT (VM), and vitrification + Mdivi-1 (VMD).

After *in vitro* culture of mouse GV oocytes for 12 h, the expression level of TOMM20 in MII oocytes was lower (*P* < *0.05*) in the vitrification group than in the fresh group (Figures 4A,B). When melatonin or Mdivi-1 were added, expression level of TOMM20 was higher (*P* < *0.05*) than in the vitrification group, but lower (*P* < *0.05*) than in fresh group. Gene expressions of *Sirt1, Pgc-1*α and *Tfam* in MII oocytes were lower (*P* < *0.05*) in the vitrification group than in the fresh group (Figure 4C). However, when melatonin or Mdivi-1 were added, expression of these three genes were increased (*P* < *0.05*) and were similar (*P* > *0.05*) to that of the fresh group, except *Tfam* expression after Mdivi-1 treatment.

Similarly, after *in vitro* culture of mouse GV oocytes for 12 h, both MMP (Figures 4D,E) and ATP content (Figure 4F) in MII oocytes were lower (*P* < *0.05*) in the vitrification group than in the fresh group. However, when melatonin or Mdivi-1 were added, MMP and ATP contents were higher (*P* < *0.05*) than in the corresponding vitrification group, with not difference (*P* > *0.05*) from the fresh group.

### Melatonin Reduces Oxidative Stress and Apoptosis Caused by Cryopreservation of Mouse GV Oocytes by Regulating p-Drp1 Ser616

**Figure 5:**
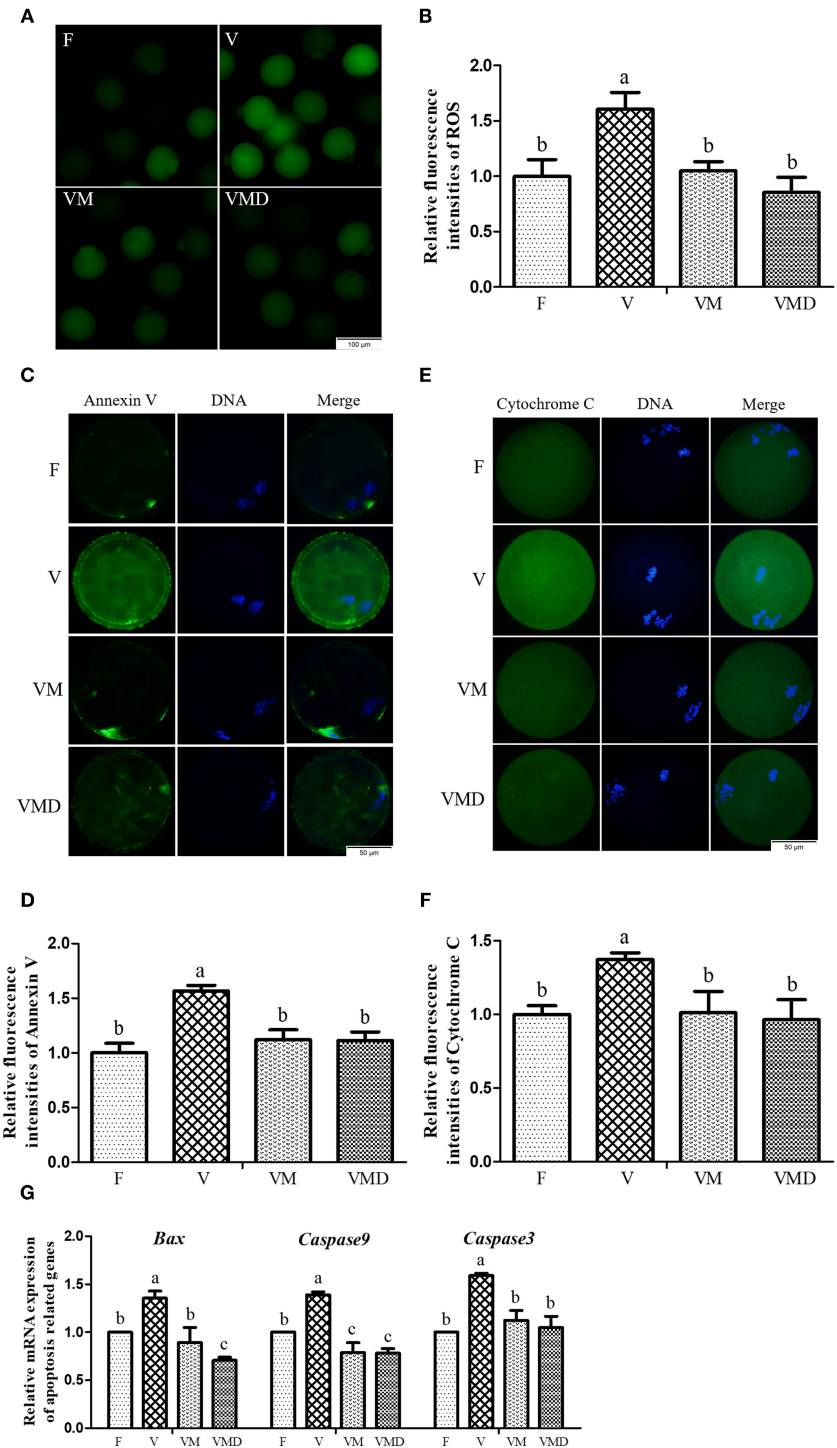
Effects of melatonin on oxidative stress and apoptosis in MII oocytes derived from vitrified-warmed mouse GV oocytes. **(A)** Representative images of oocytes staining with DCHFDA. Scale bar, 100 μm. **(B)** The fluorescence intensity of ROS levels was recorded using ImageJ software. In total, *n* = 150 MII oocytes were used in this assay (F: *n* = 32; V: *n* = 41; VM: *n* = 42; VMD: *n* = 35). **(C)** Representative images of oocytes staining with Annexin V. Scale bar, 50 μm. **(D)** The fluorescence intensity of Annexin V was recorded using ImageJ software. In total, *n* = 145 MII oocytes were used in this assay (F: *n* = 41; V: *n* = 37; VM: *n* = 36; VMD: *n* = 31). **(E)** Representative images of Cytochrome C in mouse MII oocytes. Scale bar, 50 μm. **(F)** The fluorescence intensity of Cytochrome C was recorded using ImageJ software. In total, *n* = 172 MII oocytes were used in this assay (F: *n* = 40; V: *n* = 39; VM: *n* = 46; VMD: *n* = 47). **(G)** The expression levels of pro-apoptosis related genes. Data are presented as mean ± SEM of three independent experiments. Groups without a common superscript are significantly different (*P* < *0.05*).The four experimental groups: Fresh control (F), vitrification (V), vitrification + MT (VM), and vitrification + Mdivi-1 (VMD).

After *in vitro* culture of mouse GV oocytes for 12 h, the ROS level in MII oocytes was higher (*P* < *0.05*) in the vitrification group compared to the fresh group (Figures 5A,B). However, when melatonin or Mdivi-1 were added, the ROS level was lower (*P* < *0.05*) than in the corresponding vitrification group, and not different (*P* > *0.05*) from the fresh group. Furthermore, incidence of early apoptosis (Figures 5C,D) and Cytochrome C content (Figures 5E,F) were higher (*P* < *0.05*) in the vitrification group than in the fresh group. However, when melatonin or Mdivi-1 were added, the early apoptosis level and Cytochrome C content were lower (*P* < *0.05*) than in the corresponding vitrification group, and not different (*P* > *0.05*) from the fresh group. Gene expressions of *Bax, Caspase9* and *Caspase3* were higher (*P* < *0.05*) in the vitrification group than in the fresh group (Figure 5G). When melatonin or Mdivi-1 were added, gene expressions were reduced (*P* < *0.05*): the expression of *Caspase3* was not different (*P* > *0.05*) from the fresh group, and the expression of *Caspase9* was lower (*P* < *0.05*) than in the fresh group. However, the expression of *Bax* was lower (*P* < *0.05*) compared to the fresh group after treatment of Mdivi-1, but its expression was not different (*P* > *0.05*) from the fresh group after melatonin treatment.

## Discussion

In the present study, the expression level of Drp1 did not change during *in vitro* maturation of vitrified-warmed mouse GV oocytes, whereas the level of p-Drp1 Ser616 was significantly increased at M II stage of vitrified oocytes. These results were consistent with changes caused by other stress factors. For instance, p-Drp1 Ser616 was significantly increased when rat hearts were preserved at 4°C (43) or neonatal rat cardiomyocytes were treated with high (33 mM) glucose (44). In this study and previous research, the abnormal level of p-Drp1 Ser616 was restored to the level of the control group by adding melatonin or Mdivi-1, improving *in vitro* maturation rate and parthenogenetic development of oocytes to the blastocyst stage of vitrified-warmed mouse GV oocytes (in this study), effectively protecting the rat heart from mechanical trauma-induced cardiac dysfunction (23), and protecting the mouse cardiac microvascular endothelial cells from ischemia/reperfusion injury (34). Melatonin regulated the level of p-Drp1 Ser616 to alleviate stress-induced damage, promoting cell development and functional recovery.

Cryopreservation has been implicated in mitochondrial damage and reduced vitality (2, 14, 38, 45). TOMM20 is a marker of mitochondrial (46, 47). In the present study, TOMM20 expression in MII oocytes was significantly decreased after cryopreservation of mouse GV oocytes. Similarly, this alteration of TOMM20 expression also occurred in porcine MII oocytes derived from vitrified-warmed GV oocytes (16). Furthermore, this disorder of TOMM20 was restored by addition of melatonin or Mdivi-1, suggesting that melatonin can reduce cryopreservation-induced mitochondrial damage by regulating p-Drp1 Ser616.

Mitochondrial biogenesis is the embodiment of mitochondrial function, with an important role in maintaining mitochondrial homeostasis (48, 49). Cryopreservation altered biogenesis-related expression of mitochondrial genes. For example, expressions of *Sirt1, Pgc-1*α and *Tfam* genes were significantly decreased after cryopreservation of porcine GV oocytes (16) and 2-cell mouse embryos (15). In the present study, a similar phenomenon was also observed after cryopreservation of mouse GV oocytes. Sirt1 mediated activation of Pgc-1α through Pgc-1α deacetylation (50, 51), potentially regulating mitochondrial biogenesis and mitochondrial metabolism (52). Meanwhile, Pgc-1α could also activate Tfam, which is required for mtDNA transcription and mtDNA replication, through co-activation of nuclear respiratory factor 1 (Nrf1) (53). Disordered expression of genes affects mitochondrial ATP synthesis through Sirt1-Pgc-1α or Sirt1-Pgc-1α-Nrf1-Tfam pathways (54, 55). Therefore, it seems that in the present study, abnormal expression of *Sirt1, Pgc-1*α and *Tfam* in mouse MII oocytes derived from vitrified-warmed GV oocytes could decrease concentrations of MMP and ATP, potentially leading to imbalances in energy metabolism. Furthermore, abnormalities in the expression of genes *Sirt1, Pgc-1*α and *Tfam*, MMP and ATP content were corrected after addition of melatonin or Mdivi-1, suggesting that melatonin can improve mitochondrial function and maintain mitochondrial energy metabolism by regulating p-Drp1 Ser616 during *in vitro* development of vitrified-warmed mouse GV oocytes. Thereby, it promoted *in vitro* maturation of mouse GV oocytes and their subsequent embryonic development after parthenogenetic activation.

Mitochondria are the major energetic sites of the cell, producing ATP through oxidative phosphorylation. While producing ATP, mitochondria are also the main source of ROS (56, 57). In the present study, excessive ROS production induced by the vitrification of mouse GV oocytes appeared in mouse MII oocytes during *in vitro* maturation. This was also observed in previous reports from our laboratory (2) and others (58). Excessive ROS impairs mitochondrial function (59). Mitochondrial damage increased the opening of mitochondrial permeability transition pore (MPTP), leading to the outflow of Cytochrome C from intermembrane spaces. Cytochrome C binds to deoxyadenosine triphosphate (dATP) and apoptotic protease activating factor 1 (Apaf-1) to form an apoptosome, and then activated procaspase 9 initiates the apoptosis program (60). Therefore, in the present study, increased Cytochrome C level in mouse MII oocytes could have increased early apoptosis after cryopreservation of mouse GV oocytes. However, the release of Cytochrome C and the apoptosis of MII oocytes developed from vitrified-warmed mouse GV oocytes were decreased after supplementation of melatonin or Mdivi-1 in the warming and maturation solutions. These beneficial effects of melatonin were also observed in somatic cells. Melatonin relieved PrP^106−126^-induced apoptosis mouse N2a cells (35), reduced mechanical damage-induced apoptosis in H9C2 rat cardiomyocytes (23), and protected human SH-SY5Y cells from rotenone-induced damage (33) via reduction of the release of Cytochrome C. In addition, melatonin or Mdivi-1 alleviated apoptosis in mouse MII oocytes derived from the vitrified-warmed GV oocytes by reducing expression of pro-apoptosis related genes (*Bax, Caspase9, Caspase3*) in the present study, thereby promoting *in vitro* development of vitrified-warmed GV oocytes.

## Conclusions

Cryopreservation of mouse GV oocytes resulted in a high level of p-Drp1 Ser616, a low level of mitochondrial mass marker-TOMM20, and the mitochondrial dysfunction that was characterized by alterations in MMP, ATP and expression of *Sirt1, Pgc-1*α and *Tfam* genes. Cryopreservation also increased ROS and apoptosis levels in MII oocytes and decreased their *in vitro* developmental potential. However, addition of 10^−7^mol /L melatonin restored the mitochondrial function, reduced oxidative stress, decreased the expression of pro-apoptosis-related genes (i.e., *Bax, Caspase9, Caspase3*) and the release of Cytochrome C, and inhibited apoptosis by regulating p-Drp1 Ser616. Collectively, these ameliorative effects of melatonin resulted in the improved *in vitro* maturation of vitrified-warmed mouse GV oocytes and their subsequent development to blastocysts after parthenogenetic activation.

## Data Availability Statement

The original contributions presented in the study are included in the article/supplementary material, further inquiries can be directed to the corresponding author/s.

## Ethics Statement

The animal study was reviewed and approved by Animal Ethical and Welfare Committee (AEWC) of Sichuan Agricultural University.

## Author Contributions

JQ and GZ conceptualization, visualization, and validation. JQ, SG, JY, BP, SZ, and TL methodology. SG and JY formal analysis. JQ investigation, data curation, and writing—original draft preparation. GZ and YF resources. GZ, YF, and IQ writing—review and editing. GZ supervision, project administration, and funding acquisition. All authors have read and agreed to the published version of the manuscript.

## Funding

This research was funded by the Natural Science Found of Qinghai Province (2020-ZJ-902) and the Strategic Priority Research Program of the Chinese Academy of Sciences (XDA27040302).

## Conflict of Interest

The authors declare that the research was conducted in the absence of any commercial or financial relationships that could be construed as a potential conflict of interest.

## Publisher's Note

All claims expressed in this article are solely those of the authors and do not necessarily represent those of their affiliated organizations, or those of the publisher, the editors and the reviewers. Any product that may be evaluated in this article, or claim that may be made by its manufacturer, is not guaranteed or endorsed by the publisher.
